# Can the prevalence of one STI serve as a predictor for another? A mathematical modeling analysis

**DOI:** 10.1016/j.idm.2024.12.008

**Published:** 2024-12-12

**Authors:** Ryosuke Omori, Hiam Chemaitelly, Laith J. Abu-Raddad

**Affiliations:** a Division of Bioinformatics, International Institute for Zoonosis Control, Hokkaido University, Sapporo, Hokkaido, Japan; b Infectious Disease Epidemiology Group, Weill Cornell Medicine-Qatar, Cornell University, Doha, Qatar; c World Health Organization Collaborating Centre for Disease Epidemiology Analytics on HIV/AIDS, Sexually Transmitted Infections, and Viral Hepatitis, Weill Cornell Medicine–Qatar, Cornell University, Qatar Foundation – Education City, Doha, Qatar; d Department of Population Health Sciences, Weill Cornell Medicine, Cornell University, New York, NY, USA; e Department of Public Health, College of Health Sciences, QU Health, Qatar University, Doha, Qatar; f College of Health and Life Sciences, Hamad bin Khalifa University, Doha, Qatar

**Keywords:** Sexually transmitted infection, Sexually transmitted disease, Men who have sex with men, Mathematical modeling, Epidemiology, Public health

## Abstract

We aimed to understand to what extent knowledge of the prevalence of one sexually transmitted infection (STI) can predict the prevalence of another STI, with application for men who have sex with men (MSM). An individual-based simulation model was used to study the concurrent transmission of HIV, HSV-2, chlamydia, gonorrhea, and syphilis in MSM sexual networks. Using the model outputs, 15 multiple linear regression models were conducted for each STI prevalence, treating the prevalence of each as the dependent variable and the prevalences of up to four other STIs as independent variables in various combinations. For HIV, HSV-2, chlamydia, gonorrhea, and syphilis, the proportion of variation in prevalence explained by the 15 models ranged from 34.2% to 88.3%, 19.5%–70.5%, 43.7%–82.9%, 48.7%–86.3%, and 19.5%–67.2%, respectively. Including multiple STI prevalences as independent variables enhanced the models' predictive power. Gonorrhea prevalence was a strong predictor of HIV prevalence, while HSV-2 and syphilis prevalences were weak predictors of each other. Propagation of STIs in sexual networks reveals intricate dynamics, displaying varied epidemiological profiles while also demonstrating how the shared mode of transmission creates ecological associations that facilitate predictive relationships between STI prevalences.

## 1 Introduction

Sexually transmitted infections (STIs) share a common mode of transmission, resulting in overlapping epidemiologies among them (Kouyoumjian et al., 2018; Low et al., 2006; Omori & Abu-Raddad, 2017; Omori et al., 2024). However, these infections exhibit distinct biological profiles, including variations in natural history, transmissibility, immune responses, and potential for spontaneous resolution or curability through treatment (Brunham & Plummer, 1990; Low et al., 2006; Omori et al., 2024). These differences affect how STIs spread through various types of sexual networks (Omori & Abu-Raddad, 2017; Omori et al., 2024). Specific network structures may preferentially facilitate the transmission of certain STIs, but not others (Omori & Abu-Raddad, 2017). Accordingly, the prevalence of different STIs varies depending on sexual network characteristics and is influenced by nuanced threshold and saturation effects (Omori & Abu-Raddad, 2017; Omori et al., 2024).

We aimed to address the following question: To what extent can knowledge of the prevalence of one STI in a given population predict the prevalence of another STI within the same population? This question holds both theoretical and practical significance. Theoretically, it is important to determine the extent to which the shared mode of transmission among STIs facilitates predictive relationships regarding their prevalences. Practically, understanding these relationships informs public health responses, enabling more effective interventions. To explore this, we simulated STI epidemics across diverse sexual networks, focusing on men who have sex with men (MSM), a population disproportionately affected by STIs (Brunham & Plummer, 1990).

## 2 Methods

An individual-based Monte Carlo simulation model was used to simulate diverse sexual networks among MSM and the concurrent transmission of HIV, herpes simplex virus type 2 (HSV-2), chlamydia, gonorrhea, and syphilis. Details regarding the model's structure, parameterization, sexual network simulation with varying network statistics, and infection transmission dynamics have been previously reported (Omori & Abu-Raddad, 2017; Omori et al., 2024) and are briefly summarized below. The simulations were implemented using the C programming language, and statistical analyses were conducted in R version 4.1.2.

The model simulated sexual partnerships, including both long-term and short-term relationships, along with birth, death, and infection transmission within each sexual network (Omori & Abu-Raddad, 2017; Omori et al., 2024). While the model included individuals from all age groups, only those aged 15–64 were considered sexually active. Infected individuals progressed through the natural history stages of each STI assuming an exponential distribution, where the rate corresponded to the inverse of each stage's duration.

Simulations were initiated with a population of 2000 individuals. To maintain the cohort size, individuals who died were replaced by newborns without any STI history, who would age and eventually reenter the sexual network. To prevent stochastic extinction in the finite simulated network, an average of one infection per STI was randomly introduced each year.

The mean and variance of the number of partners within the past year, for both long-term and short-term partnerships, as well as the degree correlation across short-term and all partnerships, the clustering coefficient for short-term partnerships, and the prevalence of concurrency in short-term and all partnerships were varied across diverse ranges (Omori & Abu-Raddad, 2017; Omori et al., 2024). Definitions and derivations of these network statistics within sexual networks have been previously reported (Omori & Abu-Raddad, 2017). A total of 500 distinct sexual networks were generated to represent the global variation in MSM sexual networks (Omori et al., 2024).

The probability of STI transmission within a sexual partnership was calculated using the binomial model (Chemaitelly et al., 2022; Rottingen & Garnett, 2002), which accounted for the STI transmission probability per sexual act and the total number of acts within the partnership. The partner change rate and frequency of sexual acts were age-dependent, informed by sexual behavior data (Choi et al., 2010; Weinstein et al., 1990). Infection transmission occurred exclusively through anal sex. No biological interactions between the STIs were assumed, so each propagated independently within the network. To establish endemic equilibrium in the simulations, a burn-in period of 200 years was implemented. Analyses were conducted at this endemic equilibrium to disentangle epidemiological effects from temporal variations.

The model parameters, listed in Table S1 with their respective references, were chosen based on current knowledge of the natural history, transmission, immune response, treatment, and epidemiology of each considered STI (Johnson & Geffen, 2016; Omori & Abu-Raddad, 2017; Omori et al., 2024). A detailed discussion of these parameters is provided in Omori et al. (Omori et al., 2024). These parameters characterize the attributes of each STI, including the structure of infection stages, durations of each stage, shedding frequency, transmission probability per sexual act categorized by infection stage, proportion of symptomatic versus asymptomatic infections, proportion of infections effectively treated, proportion of infections conferring immunity post-treatment or through natural clearance, and duration of immunity.

Multiple linear regression analyses were conducted for each STI prevalence, with the considered STI prevalence as a continuous dependent variable, and the prevalences of other STIs as continuous independent variables. For each STI, 15 regression models were constructed to investigate the effect of each other STI's prevalence, either individually or in combinations of two, three, or four. These models were used to estimate the standardized partial regression coefficients, which represent the effect sizes (ESs) of the independent variables in the regression equations. The ESs were categorized as weak, intermediate, strong, and very strong if the ES was <0.5, 0.5 to <0.7, 0.7 to <0.9, and ≥0.9, respectively. Bootstrap resampling, with 1000 iterations, was employed to derive estimates of the 95% confidence intervals for the ESs. Further details on this statistical analysis approach can be found in Omori et al. (Omori & Abu-Raddad, 2017).

## 3 Results

**Fig. 1:**
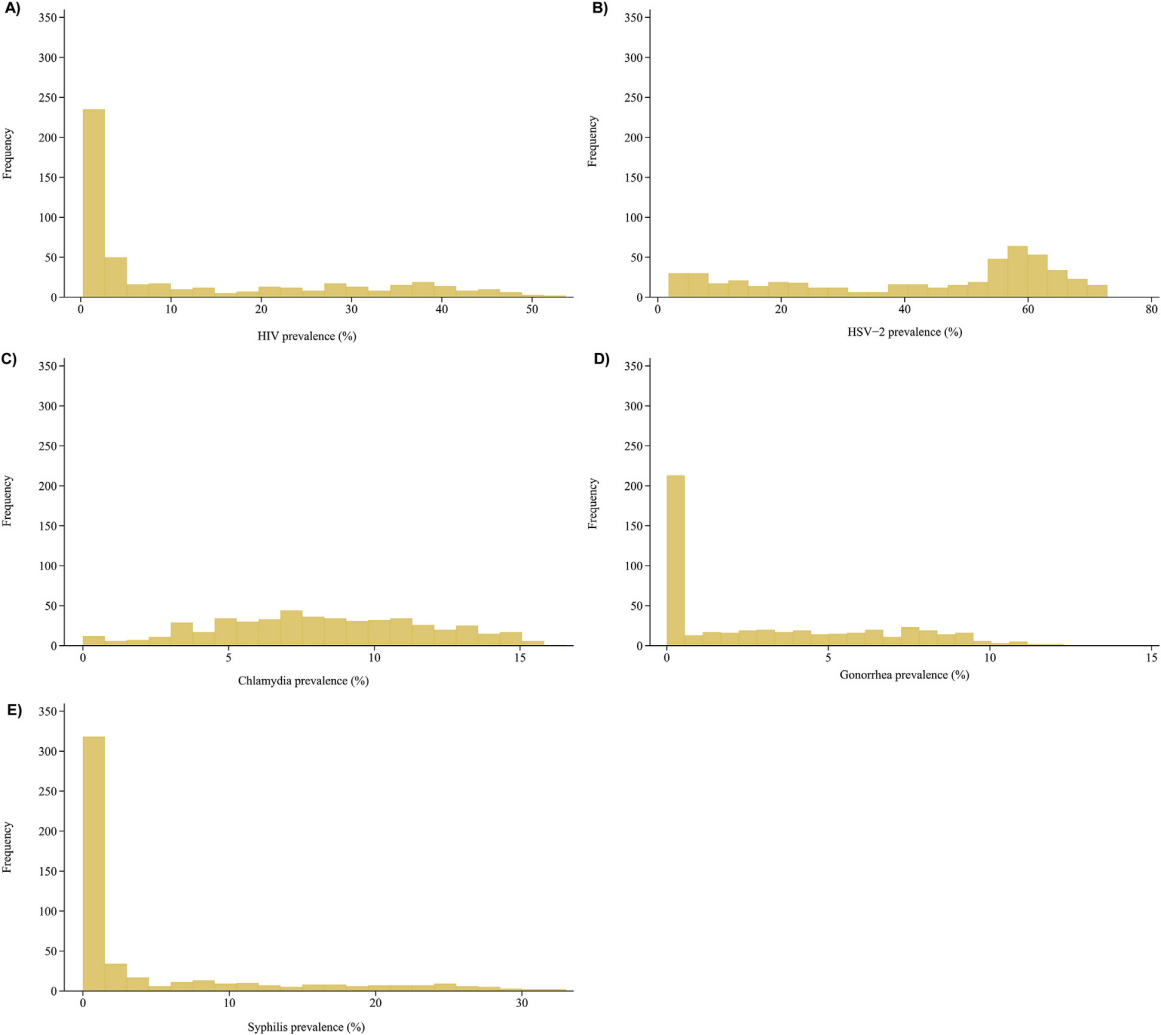
Distribution of the simulated STI prevalence across the 500 simulated STI epidemics in diverse MSM sexual networks for HIV (A), HSV-2 (B), chlamydia (C), gonorrhea (D), and syphilis (E).

**Fig. 2:**
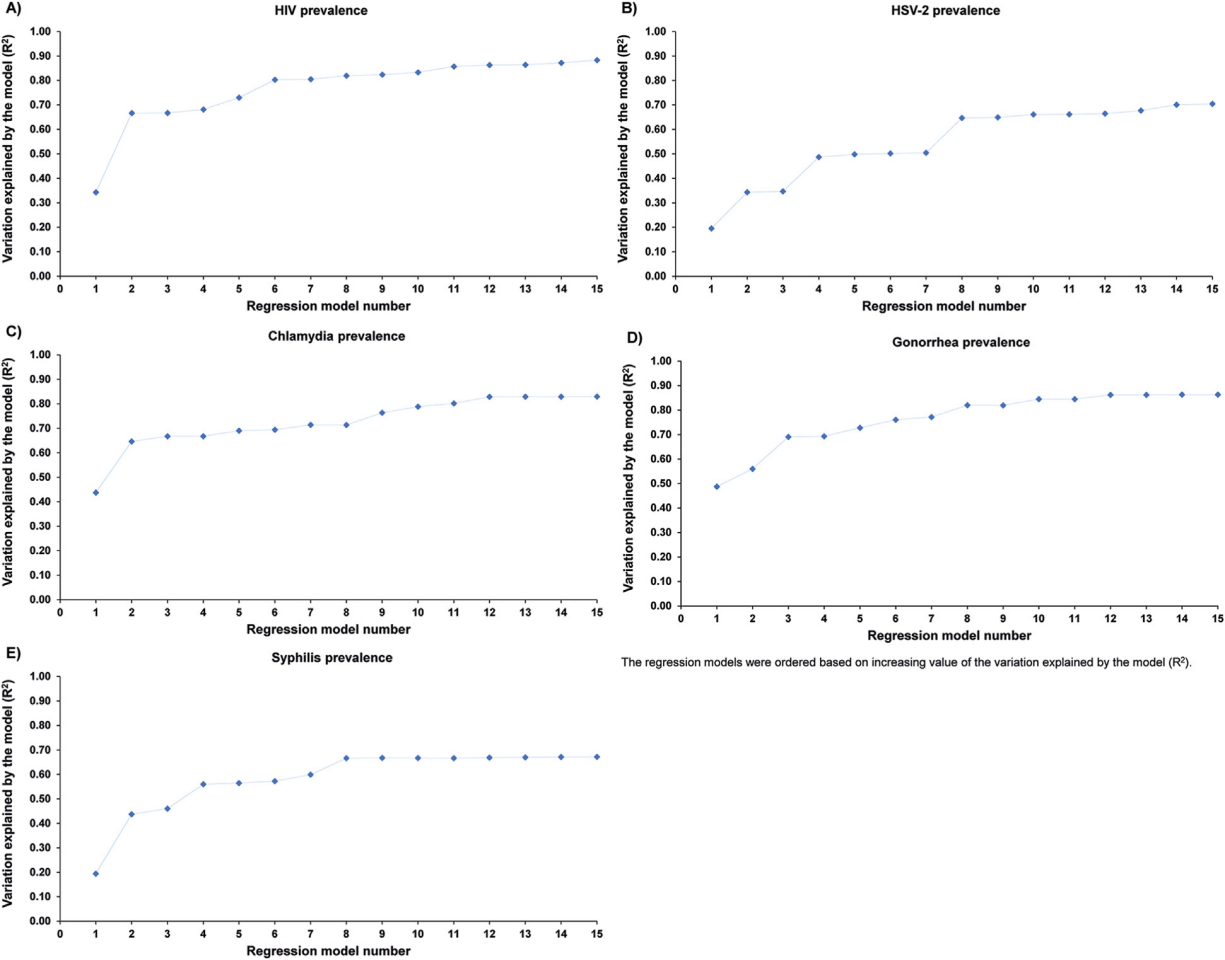
Proportion of the variation explained for each of HIV (A), HSV-2 (B), chlamydia (C), gonorrhea (D), and syphilis (E) prevalence by the other STI prevalences in the 15 regression models. The models include the other STI prevalences as independent variables individually or in combinations of two, three, or four (Table S2). The models in each panel are ordered based on increasing variation explained.

Fig. 1 shows the distribution of the simulated STI prevalence across the 500 simulated STI epidemics in diverse MSM sexual networks. Table S2 displays the estimated ESs across the set of 15 models for each STI prevalence. Fig. 2 illustrates, for each considered STI prevalence, the proportion of variation explained by the other STI prevalences in each model. Including additional STI prevalences as independent variables in the prediction models—specifically in combinations of two, three, or four—enhanced the models' predictive ability, accounting for a larger proportion of the variation in prevalence.

For HIV prevalence, the proportion of the variation explained across the 15 models ranged from 34.2% to 88.3% (Table S2). For HSV-2, chlamydia, gonorrhea, and syphilis, the ranges were 19.5%–70.5%, 43.7%–82.9%, 48.7%–86.3%, and 19.5%–67.2%, respectively.

In the subset of models where only one STI prevalence was included as a predictor (Table S3), the ES of this STI prevalence on the predicted STI prevalence was consistently positive. The ES exceeded 0.4, indicating an appreciable predictive power, and the proportion of variation explained surpassed 19%. Each STI prevalence contributed to the prediction of other STI prevalences, at least to some extent and explained a portion of their observed variation. However, the predictability varied among the different STIs.

The order of increasing predictability for HIV prevalence was as follows: HSV-2, syphilis, chlamydia, and gonorrhea (Table S3). For HSV-2 prevalence, the sequence was syphilis, HIV, gonorrhea, and chlamydia. For chlamydia prevalence, it was syphilis, HSV-2, HIV, and gonorrhea. For gonorrhea prevalence, it was HSV-2, syphilis, chlamydia, and HIV. Finally, for syphilis prevalence, it was HSV-2, chlamydia, gonorrhea, and HIV. Notably, gonorrhea prevalence alone was a strong predictor of HIV prevalence, while HSV-2 and syphilis prevalences were weak predictors of each other.

In the models that included combinations of two, three, or four STI prevalences, some of the ESs were negative (Table S2). In most cases, this negative ES pertained to the effects of HSV-2 and syphilis prevalences as predictors when other STI prevalences were also included in the prediction model.

## 4 Discussion

Even though sexually transmitted pathogens propagate largely independently within sexual networks and exhibit stark differences in their biology, their shared mode of transmission creates ecological associations between any two STI prevalences. These associations make one STI prevalence predictive of another, at least to some extent. Knowledge of an STI prevalence in a population is a useful data point that can be used to predict the prevalence of another STI. Knowledge of several STI prevalences enhances the ability to predict the prevalence of another STI. Most of the variation observed in a specific STI prevalence across populations can be explained by the prevalences of a few other STIs. These findings affirm the limitations of attributing associations between different STIs to biological causal factors (Omori et al., 2018).

These findings illustrate that any given STI prevalence is, in reality, a proxy summary measure of certain aspects of sexual behavior, leading to syndemicity among STIs. This syndemicity demonstrates the importance of a holistic approach to managing STIs collectively, rather than in isolation. This syndemicity presents also an opportunity, as addressing any one STI is almost certain to have an impact on others.

Yet, the results also reveal a spectrum of differences among STIs. HSV-2 and syphilis were the weakest predictors of other STIs because they represent extremes in STI epidemiology (Omori et al., 2024). HSV-2, which easily propagates within sexual networks, can infect a large proportion of the population, including those at lower sexual risk. Conversely, syphilis tends to concentrate among a small proportion of the population exhibiting the highest sexual risk behaviors. Due to this “fringe STI epidemiology” of these two infections, the ESs of their prevalences on predicting other STI prevalences are relatively small and can even be negative when other STI prevalences are included in the prediction model. This negative ES arises because certain features of sexual networks, such as clustering, can facilitate the propagation of some STIs like the weakly transmissible HIV by sustaining its transmission chains, while simultaneously limiting the spread of highly transmissible STIs like HSV-2 to more remote areas of the sexual network (Omori & Abu-Raddad, 2017).

This study has limitations. The results were generated based on current understanding of the biologies of the five STIs, some of which are still not fully understood. The model simulated STI transmission exclusively through anal sex, although some STIs can also be transmitted via other sexual and non-sexual routes. The study did not account for transmission links involving non-male partners or the broader population beyond MSM. We also assumed no biological interactions between STIs, despite observational evidence suggesting that such interactions may occur (Korenromp et al., 2001; Looker et al., 2017; Omori et al., 2018). Lastly, the study did not examine the predictive value of a history of one STI for the current prevalence of another, an area with potential subtle effects that warrants future research.

This study highlighted the intricate dynamics among STIs within sexual networks, revealing not only varied epidemiological profiles but also how the shared mode of transmission creates ecological associations that facilitate predictive relationships between STI prevalences. These relationships support a holistic approach to STI management, where addressing one infection can positively impact the control of others and optimize public health interventions.

## CRediT authorship contribution statement

**Ryosuke Omori:** Writing – review & editing, Writing – original draft, Validation, Software, Methodology, Formal analysis, Conceptualization, Project administration. **Hiam Chemaitelly:** Writing – review & editing, Writing – original draft, Formal analysis. **Laith J. Abu-Raddad:** Writing – review & editing, Writing – original draft, Supervision, Project administration, Funding acquisition, Conceptualization.

## Patient consent for publication

Not required.

## Data availability statement

All relevant data are within the manuscript and its Supplementary Material. The model's C programming language code is available at: https://github.com/ryosukeomori/share_msm_temporal_net_fivestis/blob/main/src/.

## Ethics approval

Not required as this is a mathematical modeling study.

## Funding

RO acknowledges the support of 10.13039/501100009023Precursory Research for Embryonic Science and Technology (PRESTO) grant number JPMJPR15E1 from 10.13039/501100002241Japan Science and Technology Agency, Japan Society for the Promotion of Science (JSPS), Grant-in-Aid for Young Scientists (B) 19K20393, and Japan Agency for Medical Research and Development (AMED) under Grant Number JP23fk0108676. Research reported in this publication was supported by the Qatar Research Development and Innovation Council [ARG01-0522-230273]. The content is solely the responsibility of the authors and does not necessarily represent the official views of Qatar Research Development and Innovation Council. The funders had no role in study design, data collection and analysis, decision to publish, or preparation of the manuscript. The authors are also grateful for infrastructure support provided by the Biostatistics, Epidemiology, and Biomathematics Research Core at Weill Cornell Medicine-Qatar.

## Declaration of competing interest

The authors declare that they have no known competing financial interests or personal relationships that could have appeared to influence the work reported in this paper.
