# Supplementary material for: Can the prevalence of one STI serve as a predictor for another? A mathematical modeling analysis
Source: Infect Dis Model. 2024 Dec 12;10(2):423–8. doi: 10.1016/j.idm.2024.12.008 (PMC11729652; doi:10.1016/j.idm.2024.12.008)
Supplement: Multimedia component 1 [file mmc1.docx]

**Supplementary Material**

**Table S1. Model parameters, as compiled for the used model.(Omori et al., 2024)**

| **Parameters** | **Value** | **Sources** |
| --- | --- | --- |
| **HIV** | | |
| HIV transmission probability per vaginal sex act |  |  |
| Acute infection | 0.0360 | Published literature (Hollingsworth et al., 2008; Pinkerton, 2008; Wawer et al., 2005) |
| Chronic infection | 0.0008 | Published literature (Hollingsworth et al., 2008; Pinkerton, 2008; Wawer et al., 2005) |
| Advanced infection | 0.0042 | Published literature (Hollingsworth et al., 2008; Pinkerton, 2008; Wawer et al., 2005) |
| HIV cofactor for anal sex transmission^*^ | 1.5 | Published literature (Baggaley et al., 2010; Patel et al., 2014) |
| Duration of each HIV stage |  |  |
| From acute to chronic | 49 days | Published literature (Abu-Raddad & Longini, 2008; Pinkerton, 2008; Wawer et al., 2005) |
| From chronic to AIDS | 9.0 years | Published literature (Abu-Raddad & Longini, 2008; Morgan & Whitworth, 2001; UNAIDS, 2007) |
| From AIDS to death | 2.0 years | Published literature (Abu-Raddad & Longini, 2008; Wawer et al., 2005) |
| **HSV-2** |  |  |
| HSV-2 transmission probability per anal sex act |  |  |
| Primary infection | 0.004 | Published literature (Abu-Raddad et al., 2008) |
| Latent infection | 0.0 | Published literature (Abu-Raddad et al., 2008) |
| Reactivation | 0.004 | Published literature (Abu-Raddad et al., 2008) |
| Duration of each HSV-2 stage |  |  |
| From primary to latent | 20 days | Published literature (Abu-Raddad et al., 2008) |
| From latent to reactivation | 78.5 days | Published literature (Abu-Raddad et al., 2008) |
| From reactivation to latent | 12.8 days | Published literature (Abu-Raddad et al., 2008) |
| HSV-2 shedding frequency | 14% | Published literature (Abu-Raddad et al., 2008; Mark et al., 2008) |
| **Chlamydia** |  |  |
| Chlamydia transmission probability per anal sex act | 0.17 | Published literature (Jenness et al., 2017; Xiridou et al., 2013) |
| Proportion of chlamydia infections |  |  |
| Becoming symptomatic among males | 0.30 | Published literature (Johnson & Geffen, 2016) |
| Successfully treated | 0.70 | Published literature (Johnson & Geffen, 2016) |
| Immune after treatment | 0.5 | Published literature (Johnson & Geffen, 2016) |
| Duration of |  |  |
| Symptomatic chlamydia infection | 16 weeks | Published literature (Johnson & Geffen, 2016) |
| Asymptomatic chlamydia infection | 90 weeks | Published literature (Johnson & Geffen, 2016) |
| Immunity | 520 weeks | Published literature (Johnson & Geffen, 2016) |
| **Gonorrhea** |  |  |
| Gonorrhea transmission probability per anal sex act | 0.46 | Published literature (Zhang et al., 2017) |
| Proportion of gonorrhea infections |  |  |
| Becoming symptomatic among males | 0.64 | Published literature (Johnson & Geffen, 2016; Newman et al., 2015; Rowley et al., 2019) |
| Successfully treated | 0.70 | Published literature (Johnson & Geffen, 2016) |
| Immune after treatment | 0.5 | Published literature (Johnson & Geffen, 2016) |
| Duration of |  |  |
| Symptomatic or asymptomatic infection if untreated | 20 weeks | Published literature (Johnson & Geffen, 2016) |
| Immunity | 52 weeks | Published literature (Johnson & Geffen, 2016) |
| **Syphilis** |  |  |
| Syphilis transmission probability per anal sex act | 0.20 | Average of male-to-female and female-to-male transmission probability (Johnson & Geffen, 2016) |
| Duration of |  |  |
| Incubation | 4.4 weeks | Published literature (Johnson & Geffen, 2016) |
| Primary syphilis | 6.6 weeks | Published literature (Johnson & Geffen, 2016) |
| Secondary syphilis | 15.6 weeks | Published literature (Johnson & Geffen, 2016) |
| Latent syphilis | 520 weeks | Published literature (Johnson & Geffen, 2016) |
| Proportion of |  |  |
| Primary cases seronegative after successful treatment | 0.40 | Published literature (Johnson & Geffen, 2016) |
| Primary syphilis cases successfully treated | 0.70 | Published literature (Johnson & Geffen, 2016) |
| Secondary syphilis cases successfully treated^†^ | 30% lower than for primary syphilis | Published literature^†^ (Ganesan et al., 2015; Johnson & Geffen, 2016) |
| Reduction in health seeking behavior for secondary syphilis compared to primary syphilis | 0.5 | Published literature (Johnson & Geffen, 2016) |
| Duration from |  |  |
| Recovery in early disease to seronegative susceptible | 26 weeks | Published literature (Johnson & Geffen, 2016) |
| Recovery in late disease to seronegative susceptible | 52 weeks | Published literature (Johnson & Geffen, 2016) |
| **Sexual behavior** |  |  |
| Long-term (spousal) partnership formation rate | 0.12 | Representative value informed by rate of spousal partnership formation rate among the heterosexual population (MEASURE DHS; Omori & Abu-Raddad, 2017) |
| Mean duration |  |  |
| Long-term (spousal) sexual partnership | 5 years | Representative value informed by duration of spousal partnerships among the heterosexual population (MEASURE DHS; Omori & Abu-Raddad, 2017) |
| Short-term (casual) sexual partnership | 2 weeks | Representative value and informed by previous work (Abu-Raddad & Longini Jr, 2008; Awad & Abu-Raddad, 2014) |
| Number of short-term (casual) sex partners among individuals not in long-term (spousal) partnerships over the last year |  |  |
| Mean | 0.0-5.0 | Representative range informed by analyses of empirical data (Omori et al., 2015) |
| Variance | 0.0-5.0 |  |
| Number of short-term (casual) sex partners among individuals in long-term (spousal) partnerships over the last year |  |  |
| Mean | 0.0-2.5 | Representative range informed by analyses of empirical data (Omori et al., 2015) |
| Variance | 0.0-2.5 |  |
| Tuning parameter |  |  |
| Degree correlation (*κ_corr_*) | -10.0-10.0 | Representative range to generate wide variation in degree correlation(Omori & Abu-Raddad, 2017) |
| Clustering (*κ_clus_*) | 0.0-20.0 | Representative range to generate wide variation in clustering (Omori & Abu-Raddad, 2017) |
| **Demographic factors** |  |  |
| Natural mortality rate per year by age group |  |  |
| 0-4 years | 0.04 |  |
| 5-69 years | 0.0026 |  |
| 70+ years | 0.0998 |  |

HIV denotes human immunodeficiency virus; HSV-2, herpes simplex virus type 2.

^*^The transmission probability per one anal sex act is determined as the product of HIV transmission probability per coital act for vaginal sex time the HIV cofactor for anal sex transmission.

^†^Probability of treatment success was assumed to be 30% lower for secondary syphilis than for primary syphilis, due to the greater difficulty of diagnosing secondary syphilis symptoms and the lower efficacy of penicillin in treating syphilis of longer duration (Ganesan et al., 2015; Johnson & Geffen, 2016).

**Table S2. Estimated standardized partial regression coefficients, representing the effect sizes of the prevalences of sexually transmitted infections, across the 15 regression models for each predicted infection prevalence. The 15 regression models investigate the effect of each other sexually transmitted infection prevalence, either individually or in combinations of two, three, or four.**

| **Sexually transmitted infection** | **Standardized partial regression coefficient (95% CI)** | | | | | **R^2^** |
| --- | --- | --- | --- | --- | --- | --- |
|  | **HIV** | **HSV-2** | **Chlamydia** | **Gonorrhea** | **Syphilis** |  |
| **HIV** |  | 0.586 (0.514-0.657) |  |  |  | 0.343 |
|  |  |  |  |  | 0.816 (0.766-0.867) | 0.667 |
|  |  |  | 0.817 (0.766-0.868) |  |  | 0.667 |
|  |  | -0.201 (-0.284- -0.117) | 0.978 (0.894-1.062) |  |  | 0.681 |
|  |  | 0.280 (0.229-0.331) |  |  | 0.693 (0.642-0.744) | 0.730 |
|  |  |  | 0.492 (0.440-0.544) |  | 0.491 (0.439-0.543) | 0.803 |
|  |  | -0.078 (-0.0145- -0.011) | 0.563 (0.483-0.643) |  | 0.478 (0.425-0.531) | 0.805 |
|  |  |  |  | 0.905 (0.868-0.943) |  | 0.819 |
|  |  | -0.089 (-0.141- -0.037) |  | 0.967 (0.916-1.019) |  | 0.823 |
|  |  |  | 0.209 (0.144-0.274) | 0.731 (0.667-0.796) |  | 0.833 |
|  |  | -0.264 (-0.320- -0.208) | 0.400 (0.328-0.473) | 0.757 (0.697-0.817) |  | 0.857 |
|  |  |  |  | 0.669 (0.619-0.718) | 0.316 (0.267-0.365) | 0.863 |
|  |  | -0.041 (-0.087-0.006) |  | 0.702 (0.640-0.765) | 0.309 (0.259-0.358) | 0.864 |
|  |  |  | 0.170 (0.113-0.228) | 0.538 (0.474-0.603) | 0.301 (0.253-0.349) | 0.872 |
|  |  | -0.185 (-0.238- -0.132) | 0.310 (0.242-0.378) | 0.586 (0.523-0.649) | 0.254 (0.207-0.302) | 0.883 |
| **HSV-2** |  |  |  |  | 0.441 (0.362-0.520) | 0.195 |
|  | 0.586 (0.514-0.657) |  |  |  |  | 0.343 |
|  | 0.676 (0.553-0.800) |  |  |  | -0.111 (-0.234-0.012) | 0.347 |
|  |  |  |  | 0.698 (0.635-0.761) |  | 0.487 |
|  | -0.253 (-0.400- -0.106) |  |  | 0.927 (0.780-1.074) |  | 0.498 |
|  |  |  |  | 0.835 (0.741-0.929) | -0.184 (-0.277- -0.090) | 0.502 |
|  | -0.148 (-0.316-0.020) |  |  | 0.934 (0.788-1.080) | -0.137 (-0.244- -0.029) | 0.505 |
|  |  |  | 0.804 (0.752-0.856) |  |  | 0.646 |
|  |  |  | 0.724 (0.631-0.818) | 0.096 (0.002-0.190) |  | 0.649 |
|  |  |  | 0.910 (0.842-0.979) |  | -0.161 (-0.229- -0.092) | 0.661 |
|  | -0.213 (-0.302- -0.124) |  | 0.978 (0.889-1.067) |  |  | 0.661 |
|  | -0.134 (-0.249- -0.019) |  | 0.976 (0.888-1.065) |  | -0.095 (-0.183- -0.006) | 0.664 |
|  |  |  | 0.757 (0.666-0.847) | 0.257 (0.155-0.360) | -0.252 (-0.328- -0.176) | 0.677 |
|  | -0.554 (-0.672- -0.436) |  | 0.840 (0.750-0.930) | 0.501 (0.379-0.623) |  | 0.701 |
|  | -0.467 (-0.601- -0.333) |  | 0.836 (0.746-0.926) | 0.509 (0.387-0.631) | -0.111 (-0.194- -0.028) | 0.705 |
| **Chlamydia** |  |  |  |  | 0.661 (0.595-0.727) | 0.437 |
|  |  | 0.804 (0.752-0.856) |  |  |  | 0.646 |
|  | 0.830 (0.742-0.918) |  |  |  | -0.17 (-0.105-0.071) | 0.667 |
|  | 0.817 (0.766-0.868) |  |  |  |  | 0.667 |
|  |  |  |  | 0.831 (0.782-0.880) |  | 0.690 |
|  |  |  |  | 0.764 (0.690-0.837) | 0.090 (0.017-0.164) | 0.694 |
|  | 0.382 (0.254-0.509) |  |  | 0.508 (0.397-0.619) | -0.031 (-0.112-0.051) | 0.714 |
|  | 0.358 (0.247-0.469) |  |  | 0.507 (0.396-0.618) |  | 0.714 |
|  |  | 0.636 (0.588-0.684) |  |  | 0.381 (0.333-0.428) | 0.763 |
|  |  | 0.437 (0.380-0.494) |  | 0.526 (0.469-0.583) |  | 0.788 |
|  |  | 0.465 (0.409-0.520) |  | .376 (0.300-0.451) | 0.175 (0.115-0.235) | 0.802 |
|  | 0.527 (0.481-0.572) | 0.496 (0.451-0.541) |  |  |  | 0.828 |
|  | 0.494 (0.423-0.564) | 0.498 (0.453-0.543) |  |  | 0.039 (-0.025-0.102) | 0.829 |
|  | 0.480 (0.393-0.566) | 0.480 (0.428-0.531) |  | 0.062 (-0.036-0.160) |  | 0.829 |
|  | 0.453 (0.354-0.552) | 0.483 (0.431-0.535) |  | 0.057 (-0.041-0.156) | 0.035 (-0.028-0.099) | 0.829 |
| **Gonorrhea** |  | 0.698 (0.635-0.761) |  |  |  | 0.487 |
|  |  |  |  |  | 0.748 (0.690-0.807) | 0.560 |
|  |  |  | 0.831 (0.782-0.880) |  |  | 0.690 |
|  |  | 0.084 (0.002-0.166) | 0.763 (0.681-0.846) |  |  | 0.693 |
|  |  | 0.456 (0.405-0.508) |  |  | 0.547 (0.496-0.598) | 0.728 |
|  |  |  | 0.597 (0.540-0.655) |  | 0.353 (0.296-0.411) | 0.761 |
|  | 0.182 (0.109-0.254) |  | 0.432 (0.345-0.519) |  | 0.382 (0.325-0.440) | 0.772 |
|  | 0.905 (0.868-0.943) |  |  |  |  | 0.819 |
|  | 0.883 (0.818-0.947) |  |  |  | 0.028 (-0.037-0.092) | 0.820 |
|  | 0.680 (0.620-0.741) |  | 0.275 (0.215-0.335) |  |  | 0.844 |
|  | 0.654 (0.575-0.732) |  | 0.276 (0.215-0.336) |  | 0.032 (-0.028-0.092) | 0.845 |
|  | 0.756 (0.715-0.796) | 0.255 (0.215-0.295) |  |  |  | 0.862 |
|  | 0.729 (0.671-0.787) | 0.230 (0.174-0.286) | 0.050 (-0.029-0.129) |  |  | 0.862 |
|  | 0.708 (0.645-0.771) | 0.258 (0.218-0.299) |  |  | 0.056 (0.000-0.113) | 0.863 |
|  | 0.685 (0.611-0.759) | 0.235 (0.179-0.292) | 0.046 (-0.033-0.125) |  | 0.055 (-0.002-0.111) | 0.863 |
| **Syphilis** |  | 0.441 (0.362-0.520) |  |  |  | 0.195 |
|  |  |  | 0.661 (0.595-0.727) |  |  | 0.437 |
|  |  | -0.256 (-0.365- -0.147) | 0.867 (0.758-0.976) |  |  | 0.460 |
|  |  |  |  | 0.748 (0.690-0.807) |  | 0.560 |
|  |  |  | 0.128 (0.024-0.233) | 0.642 (0.537-0.746) |  | 0.565 |
|  |  | -0.157 (-0.238- -0.077) |  | 0.858 (0.778-0.938) |  | 0.572 |
|  |  | -0.312 (-0.406- -0.218) | 0.354 (0.233-0.476) | 0.672 (0.571-0.773) |  | 0.599 |
|  | 0.830 (0.742-0.918) |  | -0.017 (-0.105-0.072) |  |  | 0.667 |
|  | 0.783 (0.659-0.907) |  | -0.036 (-0.131-0.059) | 0.069 (-0.060-0.198) |  | 0.667 |
|  | 0.770 (0.651-0.890) |  |  | 0.051 (-0.069-0.171) |  | 0.667 |
|  | 0.816 (0.766-0.867) |  |  |  |  | 0.667 |
|  | 0.849 (0.787-0.912) | -0.056 (-0.119-0.006) |  |  |  | 0.669 |
|  | 0.810 (0.720-0.900) | -0.093 (-0.181- -0.006) | 0.075 (-0.048-0.197) |  |  | 0.670 |
|  | 0.747 (0.627-0.868) | -0.091 (-0.162- -0.019) |  | 0.135 (-0.001-0.271) |  | 0.671 |
|  | 0.715 (0.581-0.848) | -0.124 (-0.216- -0.031) | 0.068 (-0.054-0.190) | 0.131 (-0.005-0.267) |  | 0.672 |

CI denotes confidence interval, and HSV-2, herpes simplex virus 2.

For each infection, the effect sizes were ordered based on increasing value of the variation explained by the model (R^2^).

**Table S3. Estimated standardized partial regression coefficients, representing the effect sizes of the prevalences of sexually transmitted infections, across the four regression models where only one infection was used as a predictor at a time.**

| **Sexually transmitted infection** | **Standardized partial regression coefficient (95% CI)** | | | | | **R^2^** |
| --- | --- | --- | --- | --- | --- | --- |
|  | **HIV** | **HSV-2** | **Chlamydia** | **Gonorrhea** | **Syphilis** |  |
| **HIV** |  | 0.586 (0.514-0.657) |  |  |  | 0.343 |
|  |  |  |  |  | 0.816 (0.766-0.867) | 0.667 |
|  |  |  | 0.817 (0.766-0.868) |  |  | 0.667 |
|  |  |  |  | 0.905 (0.868-0.943) |  | 0.819 |
| **HSV-2** |  |  |  |  | 0.441 (0.362-0.520) | 0.195 |
|  | 0.586 (0.514-0.657) |  |  |  |  | 0.343 |
|  |  |  |  | 0.698 (0.635-0.761 |  | 0.487 |
|  |  |  | 0.804 (0.752-0.856) |  |  | 0.646 |
| **Chlamydia** |  |  |  |  | 0.661 (0.595-0.727) | 0.437 |
|  |  | 0.804 (0.752-0.856) |  |  |  | 0.646 |
|  | 0.817 (0.766-0.868) |  |  |  |  | 0.667 |
|  |  |  |  | 0.831 (0.782-0.880) |  | 0.690 |
| **Gonorrhea** |  | 0.698 (0.635-0.761) |  |  |  | 0.487 |
|  |  |  |  |  | 0.748 (0.690-0.807) | 0.560 |
|  |  |  | 0.831 (0.782-0.880) |  |  | 0.690 |
|  | 0.905 (0.868-0.943) |  |  |  |  | 0.819 |
| **Syphilis** |  | 0.441 (0.362-0.520) |  |  |  | 0.195 |
|  |  |  | 0.661 (0.595-0.727) |  |  | 0.437 |
|  |  |  |  | 0.748 (0.690-0.807) |  | 0.560 |
|  | 0.816 (0.766-0.867) |  |  |  |  | 0.667 |

CI denotes confidence interval, and HSV-2, herpes simplex virus 2.

For each infection, the effect sizes were ordered based on increasing value of the variation explained by the model (R^2^).

**References**

Abu-Raddad, L. J., & Longini, I. M., Jr. (2008). No HIV stage is dominant in driving the HIV epidemic in sub-Saharan Africa. *AIDS*, *22*(9), 1055-1061. <https://doi.org/10.1097/QAD.0b013e3282f8af84>

Abu-Raddad, L. J., & Longini Jr, I. M. (2008). No HIV stage is dominant in driving the HIV epidemic in sub-Saharan Africa. *Aids*, *22*(9), 1055-1061.

Abu-Raddad, L. J., Magaret, A. S., Celum, C., Wald, A., Longini, I. M., Jr., Self, S. G., & Corey, L. (2008). Genital herpes has played a more important role than any other sexually transmitted infection in driving HIV prevalence in Africa [Research Support, N.I.H., Extramural

Research Support, Non-U.S. Gov't]. *PLoS ONE*, *3*(5), e2230. <https://doi.org/10.1371/journal.pone.0002230>

Awad, S. F., & Abu-Raddad, L. J. (2014). Could there have been substantial declines in sexual risk behavior across sub-Saharan Africa in the mid-1990s? [Peer-reviewed study]. *Epidemics*, *8*(0), 9-17. <https://doi.org/http://dx.doi.org/10.1016/j.epidem.2014.06.001>

Baggaley, R. F., White, R. G., & Boily, M. C. (2010). HIV transmission risk through anal intercourse: systematic review, meta-analysis and implications for HIV prevention. *Int J Epidemiol*, *39*(4), 1048-1063. <https://doi.org/10.1093/ije/dyq057>

Ganesan, A., Mesner, O., Okulicz, J. F., O'Bryan, T., Deiss, R. G., Lalani, T.,…Infectious Disease Clinical Research Program, H. I. V. S. T. I. W. G. (2015). A single dose of benzathine penicillin G is as effective as multiple doses of benzathine penicillin G for the treatment of HIV-infected persons with early syphilis. *Clin Infect Dis*, *60*(4), 653-660. <https://doi.org/10.1093/cid/ciu888>

Hollingsworth, T. D., Anderson, R. M., & Fraser, C. (2008). HIV-1 transmission, by stage of infection. *J Infect Dis*, *198*(5), 687-693. <https://doi.org/10.1086/590501>

Jenness, S. M., Weiss, K. M., Goodreau, S. M., Gift, T., Chesson, H., Hoover, K. W.,…Rosenberg, E. S. (2017). Incidence of Gonorrhea and Chlamydia Following Human Immunodeficiency Virus Preexposure Prophylaxis Among Men Who Have Sex With Men: A Modeling Study. *Clin Infect Dis*, *65*(5), 712-718. <https://doi.org/10.1093/cid/cix439>

Johnson, L. F., & Geffen, N. (2016). A Comparison of Two Mathematical Modeling Frameworks for Evaluating Sexually Transmitted Infection Epidemiology. *Sex Transm Dis*, *43*(3), 139-146. <https://doi.org/10.1097/OLQ.0000000000000412>

Mark, K. E., Wald, A., Magaret, A. S., Selke, S., Olin, L., Huang, M. L., & Corey, L. (2008). Rapidly cleared episodes of herpes simplex virus reactivation in immunocompetent adults. *J Infect Dis*, *198*(8), 1141-1149. <https://doi.org/10.1086/591913>

MEASURE DHS. *Demographic and health surveys*. ICF Macro. Retrieved May 19 from <http://www.measuredhs.com/>

Morgan, D., & Whitworth, J. (2001). The natural history of HIV-1 infection in Africa. *Nat Med*, *7*(2), 143-145. <http://www.ncbi.nlm.nih.gov/entrez/query.fcgi?cmd=Retrieve&db=PubMed&dopt=Citation&list_uids=11175832>

Newman, L., Rowley, J., Vander Hoorn, S., Wijesooriya, N. S., Unemo, M., Low, N.,…Temmerman, M. (2015). Global estimates of the prevalence and incidence of four curable sexually transmitted infections in 2012 based on systematic review and global reporting. *PLoS One*, *10*(12), e0143304.

Omori, R., & Abu-Raddad, L. J. (2017). Sexual network drivers of HIV and herpes simplex virus type 2 transmission. *AIDS*, *31*(12), 1721-1732. <https://doi.org/10.1097/QAD.0000000000001542>

Omori, R., Chemaitelly, H., & Abu-Raddad, L. J. (2015). Dynamics of non-cohabiting sex partnering in sub-Saharan Africa: a modelling study with implications for HIV transmission [Peer-reviewed study]. *Sex Transm Infect*, *91*(6), 451-457. <https://doi.org/10.1136/sextrans-2014-051925>

Omori, R., Chemaitelly, H., & Abu-Raddad, L. J. (2024). Understanding dynamics and overlapping epidemiologies of HIV, HSV-2, chlamydia, gonorrhea, and syphilis in sexual networks of men who have sex with men. *Front Public Health*, *12*, 1335693. <https://doi.org/10.3389/fpubh.2024.1335693>

Patel, P., Borkowf, C. B., Brooks, J. T., Lasry, A., Lansky, A., & Mermin, J. (2014). Estimating per-act HIV transmission risk: a systematic review. *AIDS*, *28*(10), 1509-1519. <https://doi.org/10.1097/QAD.0000000000000298>

Pinkerton, S. D. (2008). Probability of HIV transmission during acute infection in Rakai, Uganda. *AIDS Behav*, *12*(5), 677-684. <https://doi.org/10.1007/s10461-007-9329-1>

Rowley, J., Vander Hoorn, S., Korenromp, E., Low, N., Unemo, M., Abu-Raddad, L. J.,…Gottlieb, S. (2019). Chlamydia, gonorrhoea, trichomoniasis and syphilis: global prevalence and incidence estimates, 2016. *Bulletin of the World Health Organization*, *97*(8), 548. <https://www.ncbi.nlm.nih.gov/pmc/articles/PMC6653813/pdf/BLT.18.228486.pdf>

UNAIDS. (2007). UNAIDS Reference Group on Estimates, Modelling and Projections.

Wawer, M. J., Gray, R. H., Sewankambo, N. K., Serwadda, D., Li, X., Laeyendecker, O.,…Quinn, T. C. (2005). Rates of HIV-1 transmission per coital act, by stage of HIV-1 infection, in Rakai, Uganda. *J Infect Dis*, *191*(9), 1403-1409. <https://doi.org/10.1086/429411>

Xiridou, M., Vriend, H. J., Lugner, A. K., Wallinga, J., Fennema, J. S., Prins, J. M.,…van der Sande, M. A. (2013). Modelling the impact of chlamydia screening on the transmission of HIV among men who have sex with men. *BMC Infect Dis*, *13*, 436. <https://doi.org/10.1186/1471-2334-13-436>

Zhang, L., Regan, D. G., Chow, E. P. F., Gambhir, M., Cornelisse, V., Grulich, A.,…Fairley, C. K. (2017). Neisseria gonorrhoeae Transmission Among Men Who Have Sex With Men: An Anatomical Site-Specific Mathematical Model Evaluating the Potential Preventive Impact of Mouthwash. *Sex Transm Dis*, *44*(10), 586-592. <https://doi.org/10.1097/OLQ.0000000000000661>
